# Statin improves survival in patients with EGFR-TKI lung cancer: A nationwide population-based study

**DOI:** 10.1371/journal.pone.0171137

**Published:** 2017-02-03

**Authors:** Ming-Szu Hung, I-Chuan Chen, Chuan-Pin Lee, Ru-Jiun Huang, Pau-Chung Chen, Ying-Huang Tsai, Yao-Hsu Yang

**Affiliations:** 1 Division of Thoracic Oncology, Department of Pulmonary and Critical Care Medicine, Chang Gung Memorial Hospital, Chiayi Branch, Chiayi, Taiwan; 2 Department of Medicine, College of Medicine, Chang Gung University, Taoyuan, Taiwan; 3 Department of Respiratory Care, Chang Gung University of Science and Technology, Chiayi Campus, Chiayi, Taiwan; 4 Department of Emergency Medicine, Chang Gung Memorial Hospital, Chiayi Branch, Chiayi, Taiwan; 5 Department of Nursing, Chang Gung University of Science and Technology, Chiayi Campus, Chiayi, Taiwan; 6 Center of Excellence for Chang Gung Research Datalink, Chang Gung Memorial Hospital, Chiayi, Taiwan; 7 Institute of Occupational Medicine and Industrial Hygiene, National Taiwan University College of Public Health, Taipei, Taiwan; 8 Department of Environmental and Occupational Medicine, National Taiwan University Hospital and National Taiwan University, College of Medicine, Taipei, Taiwan; 9 Department of Respiratory Care, College of Medicine, Chang Gung University, Taoyuan, Taiwan; 10 Department of Traditional Chinese Medicine, Chang Gung Memorial Hospital, Chiayi Branch, Chiayi, Taiwan; 11 School of Traditional Chinese Medicine, College of Medicine, Chang Gung University, Taoyuan, Taiwan; University of Crete, GREECE

## Abstract

Long-term use of statins has been reported to reduce the risk of death in patients with lung cancer. This study investigated the effect of statin use among patients with lung cancer receiving epidermal growth factor receptor-tyrosine kinase inhibitor (EGFR-TKIs) therapy. A nationwide, population-based case-control study was conducted using the Taiwan National Health Insurance Research Database. From January 1, 1997 to December 31, 2012, a total of 1,707 statin and 6,828 non-statin matched lung cancer cohorts with EGFR-TKIs treatment were studied. Statin use was associated with a reduced risk of death (HR: 0.58, 95% CI: 0.54–0.62, p < 0.001). In addition, statin use was associated with a significantly longer median progression-free survival (8.3 months, 95% CI: 7.6–8.9 vs. 6.1 months, 95% CI: 6.0–6.4, p < 0.001) and median overall survival (35.5 months, 95% CI: 33.8–38.1 vs. 23.9 months, 95% CI: 23.4–24.7, p < 0.001). In conclusion, statins might potentially enhance the therapeutic effect and increase survival in patients with lung cancer receiving EGFR-TKI therapy.

## Introduction

Lung cancer is the most common cause of cancer death worldwide [1]. Most patients have advanced-stage disease at the initial diagnosis of lung cancer. To date, the prognosis of advanced lung cancer remains poor. Despite treatment, the 5-year survival rate for advanced lung cancer remains from 52% to 24% to 4% for local, regional, and distant stage diseases, respectively [2, 3]. Consequently, developing new strategies for lung cancer treatment is necessary.

Patients with non-small cell lung cancer (NSCLC) with specific mutations in the tyrosine kinase domain of the epidermal growth factor (*EGFR*) gene have favorable clinical outcomes with EGFR tyrosine kinase inhibitor (EGFR-TKI) therapy [4]. All mutations are reported in exons 18, 19, 20, and 21 of *EGFR* [5] and are most frequently found in lung adenocarcinoma [6]. *EGFR* mutations have been found in less than 10% of non-Asian patients with NSCLC [7] and in 30–50% of East Asians patients [8]. Missense mutations in exon 21 (L858R) and in-frame deletions within exon 19 are the most frequent EGFR-TKI sensitive mutations (80%) in patients with NSCLC [9]. For patients with NSCLC, both mutations are associated with favorable responses to first-line treatment with EGFR-TKIs, such as gefitinib [10], erlotinib [11], and afatinib [12] versus standard chemotherapy.

Statins are 3-hydroxy-3-methylglutaryl-coenzyme A reductase inhibitors, which potently reduce plasma cholesterol levels. Statins are the most commonly used drugs to treat hypercholesterolemia and have been proven to reduce both morbidity and mortality in cardiovascular events [13, 14]. Statins have anti-cancer effects through various pathways including inhibition of inflammation, immunomodulation, and angiogenesis [15]. In lung cancer cells, statins induce apoptosis [16], inhibit tumor growth [17], and inhibit angiogenesis [18]. Recently, the long-term use of statins was reported to reduce the risk of mortality in patients with lung cancer [19, 20]. Furthermore, statins can overcome EGFR-TKI resistance in lung cancer cells [21] and patients [22]. However, the effect of statin use in patients with lung cancer receiving EGFR-TKI therapy remains unclear.

In this study, we proposed that statins might enhance the effect of EGFR-TKIs and prolong survival in patients with lung cancer receiving EGFR-TKI therapy. A nationwide population-based study was conducted to determine the effect of statin use in patients with lung cancer receiving EGFR-TKI therapy.

## Material and methods

### Data source

The Taiwan National Health Insurance Research Database (NHIRD) was used as the data source. The NHIRD is a comprehensive health care database that covers nearly the entire 23.7 million population of this country. National Health Insurance (NHI) is a compulsory universal program for all residents in Taiwan. The databases were used to collect information on the patients’ characteristics such as sex and date of birth, and information regarding admissions and outpatient visits, including date of admission, date of discharge, dates of visits, and up to five discharge diagnoses or three outpatient visit diagnoses. The diagnoses were made in accordance with the International Classification of Diseases, Ninth Revision, Clinical Modification (ICD-9-CM) codes. The comprehensive utilization and enrollment information for all patients with “catastrophic illnesses” was also included in this database. The Ethics Review Board of Chang Gung Memorial Hospital, Chiayi Branch, Taiwan approved this study (201600067B1). The data in this study were analyzed anonymously in accordance with strict confidentially guidelines with regulations regarding personal electronic data protection. The requirement for informed consent was waived by the institution review board.

#### Study cohorts

**Fig 1:**
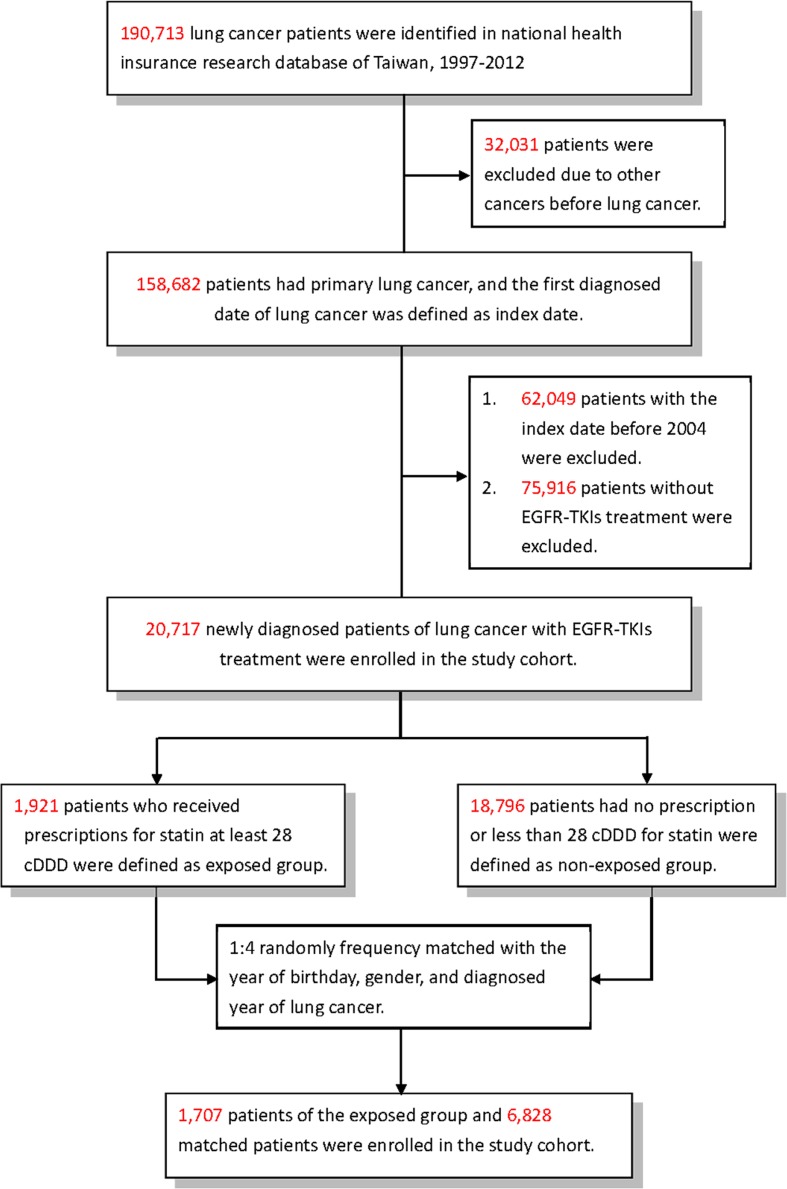
Flowchart of the patient enrollment process of statin cohort and matched non-statin cohort of patients with lung cancer EGFR-TKIs.

We identified all patients with an initial and primary diagnosis of lung cancer (ICD-9-CM 162) between January 1, 1997 and December 31, 2012 from NHIRD. Patients who were administered EGFR-TKIs (gefitinib or erlotinib) were included. Patients diagnosed with other types of cancer were excluded. The statin cohort was defined as patients who had taken statins (simvastatin, lovastatin, pravastatin, fluvastatin, atorvastatin, or rosuvastatin) for more than 28 cumulative defined daily doses after a diagnosis of lung cancer. The statin cohort was then matched in a 1:4 ratio to the non-statin cohort by year of birthday, sex, and year of lung cancer diagnosis (Fig 1). All subjects were followed up to the end of 2013.

### Demographic variables and comorbidities

Age, sex, income for estimating insurance payment, and urbanization of the subject’s residential area were included as demographic variables in this study. The patients were categorized in three levels of monthly income: ≤ NT$15,840, NT$15,841–$25,000, and ≥ NT$25,000. The urbanization level was categorized as very high, high, moderate, and low, based on population density [23]. Hypertension (ICD9-CM 401–405), diabetes mellitus (DM) (ICD9-CM 249–250), coronary artery disease (CAD) (ICD9-CM 410–419), stroke (ICD9-CM 430–438), chronic obstructive pulmonary disease (COPD) (ICD9-CM 491, 492, 496), and smoking-related disorders (ICD9-CM 305.1, 491.2, 492.8, 496, 523.6, and V15.82) were included in the baseline comorbidities for each subject. Treatments including radiotherapy (RT), chemotherapy (CT), or both were also included in our study. EGFR-TKI responders were defined as patients who were administered EGFR-TKI therapy for more than 90 days, and the remaining patients were defined as non-responders [24]. The CT regimens before EGFR-TKI therapy were also included in this study.

Overall survival (OS) was defined as the time from the diagnosis to any cause of death or when patients were censored at the last follow-up. In Taiwan, EGFR-TKIs were approved by NHI in November, 2007 (gefitinib) and June, 2008 (erlotinib) for the treatment of stage IIIB or IV lung cancer as the 2^nd^ line therapy for lung adenocarcinoma and the 3^rd^ line therapy for NSCLC (erlotinib), and in November, 2013 as the 1^st^ line therapy for lung adenocarcinoma with EGFR mutations (gefitinib and erlotinib). Every patient who received EGFR-TKI therapy was requested to undergo imaging studies and apply EGFR-TKI every three months. Once progressive disease was observed, EGFR-TKIs were declined by NHI. Since the results of imaging studies were not available in the NHIRD, we alternatively defined progression-free survival (PFS) in our study as the interval from the beginning to the end of EGFR-TKI therapy. All patients receiving 2^nd^ line gefitinib and erlotinib therapy in our study had adenocarcinoma, and those receiving 3^rd^ line erlotinib therapy had NSCLC. *EGFR* mutations in patients with lung cancer are associated with a favorable response to EGFR-TKI therapy [25]. Since *EGFR* mutation status is not available in the NHIRD, EGFR-TKI responders were used as a surrogate of *EGFR* mutations in our study.

### Propensity score-based matching

In the present study, propensity score (PS) matching was used to balance covariates in the two treatment groups[26]. The PS was the conditional probability of receiving statins treatment, as a binary dependent variable, under a set of measurements. Clinical variables including sex, age, urbanization, income, comorbidities, CT/RT, EGFR-TKI, EGFR-TKI response, and CT regimens before EGFR-TKI were added into a non-parsimonious multivariable logistic regression model to predict the risk of use of statin. The predicted probability derived from the logistic equation was used as the PS for each individual. The two groups of subjects (statin users and non-users) were combined and classified in accordance with PS, and subjects in the two groups were matched by PS. If an appropriate PS match could not be found for individual subjects within the two groups, they were excluded from further analysis. The remaining patients constituted a well-matched 1:2 prospective cohort.

### Statistical analysis

The differences in demographic characteristics and comorbidities between the statin and non-statin cohorts were examined by χ^2^ test. The hazard ratio (HR) and 95% confidence intervals (CI) of the risk of death for statin users compared with the comparison cohort was examined by Cox proportional hazard regression analysis. Survival analysis was performed with a Kaplan-Meier analysis and log-rank test. A multivariate Cox proportional hazards model was used to determine the risk factors of mortality in patients with lung cancer and their adjusted hazard ratio (aHR) within the statin cohort. All analyses were conducted using SAS statistical software (Version 9.4; SAS Institute, Cary, NC).

## Results

### Demographic characteristics and comorbidity between the statin and non-statin EGFR-TKI lung cancer cohorts

A total of 190,713 patients with lung cancer were included in our study from 1997 to 2013. After being 1:4 matched with year of birthday, sex, and year diagnosed with lung cancer, 1,707 patients were enrolled in the statin and 6,828 patients in the non-statin cohorts. The statin cohort had a significantly higher proportion of very high urbanization (p < 0.0001), monthly income <15840 NT$ and income ≥25000 NT$ (p <0.001), DM (*p* < 0.001), hypertension (p < 0.001), stroke (p < 0.001), CAD (p <0.001), and COPD (p < 0.001) compared to the non-user cohort (Table 1). The statin cohort also had significantly more patients without chemotherapy or/and radiotherapy (p < 0.001), and EGFR-TKI responders (*p* < 0.001). There was no significant difference in smoking-related disorders, EGFR-TKI (gefitinib, erlotinib, or both) usage, and CT regimens before EGFR-TKI among statin users and non-users.

**Table 1:** Characteristics of patients with EGFR-TKIs NSCLC.

	Statin				
Variables	User		Non-user		p-value
**Patients**	1,707	(100.0%)	6,828	(100.0%)	
**Sex**					1.000
Female	878	(51.4%)	3,512	(51.4%)	
Male	829	(48.6%)	3,316	(48.6%)	
**Age**					1.000
40–64	693	(40.6%)	2,772	(40.6%)	
≥65	1,014	(59.4%)	4,056	(59.4%)	
Mean (SD)	66.5	(9.6)	66.5	(9.6)	1.000
**Urbanization**					<0.001*
Very high	578	(33.9%)	1,861	(27.3%)	
High	699	(40.9%)	2,849	(41.7%)	
Moderate	277	(16.2%)	1,353	(19.8%)	
Low	153	(9.0%)	765	(11.2%)	
**Income (NT$)**					<0.001*
0	636	(37.3%)	2,279	(33.4%)	
1–15840	291	(17.0%)	1,071	(15.7%)	
15841–25000	461	(27.0%)	2,348	(34.4%)	
≥25000	319	(18.7%)	1,130	(16.5%)	
**Comorbidities**					
DM	902	(52.8%)	1,597	(23.4%)	<0.001*
Hypertension	1,338	(78.4%)	3,798	(55.6%)	<0.001*
Stroke	499	(29.2%)	1,278	(18.7%)	<0.001*
CAD	777	(45.5%)	1,744	(25.5%)	<0.001
COPD	586	(34.3%)	2,116	(31.0%)	0.008*
Smoking-related disorder	358	(21.0%)	1,365	(20.0%)	0.366
**CT/RT**					<0.001*
CT+ RT	813	(47.6%)	3,457	(50.6%)	
CT	527	(30.9%)	1,968	(28.8%)	
RT	108	(6.3%)	558	(8.2%)	
Without CT or RT	259	(15.2%)	845	(12.4%)	
**EGFR-TKI**					0.3384
Gefitinib	821	(48.1%)	3,379	(49.5%)	
Erlotinib	686	(40.2%)	2,726	(39.9%)	
Both	200	(11.7%)	723	(10.6%)	
**EGFR-TKI Response**					<0.001*
Responder	1,079	(63.2%)	3,990	(58.4%)	
Non-responder	628	(36.8%)	2,838	(41.6%)	
**CT regimens before EGFR-TKI**					0.415
≤1	1,120	(65.6%)	4,408	(64.6%)	
≥2	587	(34.4%)	2,420	(35.4%)	

“*”denotes p-value < 0.05.

### Clinical variables and HRs of death in EGFR-TKIs patients with lung cancer

The risk of death in the statin user and non-user cohorts was then compared with different clinical variables. In univariate analysis, an increased risk of death was observed in male patients (HR: 1.38, 95% CI: 1.32–1.46, p < 0.001), age ≥65 years (HR: 1.18, 95% CI: 1.12–1.24, p < 0.001), monthly income 1–15840 NT$ (HR: 1.10, 95% CI: 1.02–1.19, p = 0.012), monthly income 15841–25000 NT$ (HR: 1.09, 95% CI: 1.03–1.16, p = 0.005), DM (HR: 1.07, 95% CI: 1.01–1.13, p = 0.020), stroke (HR: 1.11, 95% CI: 1.04–1.18, p = 0.01), COPD (HR: 1.12, 95% CI: 1.06–1.18, p <0.001), smoking-related disorders (HR: 1.23, 95% CI: 1.16–1.31, p < 0.001), RT (HR: 1.34, 95% CI: 1.18–1.52, p < 0.001), and ≥2 CT regimens before EGFR-TKI therapy (HR: 1.08, 95% CI: 1.03–1.14, p = 0.002). A reduced risk of death was observed in statin users (HR: 0.61, 95% CI: 0.57–0.65, p < 0.001), those with monthly income ≥25000 NT$ (HR: 0.81, 95% CI: 0.75–0.87, p < 0.001), and EGFR-TKI responder (HR: 0.40, 95% CI: 0.38–0.42, p < 0.001) (Table 2).

**Table 2:** Comparison of HRs of death with clinical variables.

	Crude			Adjusted		
Variable	HR	95% CI	p-value	HR	95% CI	p-value
**Statin use**						
User	0.61	0.57–0.65	<0.001*	0.58	0.54–0.62	<0.001*
**Sex**						
Male	1.38	1.32–1.46	<0.001*	1.28	1.21–1.35	<0.001*
**Age**						
≥65	1.18	1.12–1.24	<0.001*	1.06	1.00–1.13	0.051
**Urbanization**						
Very high	0.88	0.81-.096	0.006*	0.93	0.85–1.03	0.160
High	0.92	0.85–1.01	0.068	0.96	0.88–1.05	0.359
Moderate	0.98	0.89–1.08	0.704	0.97	0.88–1.07	0.509
**Income (NT $)**						
1–15840	1.10	1.02–1.19	0.012*	1.04	0.96–1.12	0.333
15841–25000	1.09	1.03–1.16	0.005*	1.04	0.97–1.11	0.270
≥25000	0.81	0.75–0.87	<0.001*	0.82	0.76–0.90	<0.001
**Comorbidities**						
DM	1.07	1.01–1.13	0.020*	1.17	1.10–1.24	<0.001*
Hypertension	1.00	0.95–1.06	0.893	1.02	0.96–1.08	0.568
Stroke	1.11	1.04–1.18	0.001*	1.10	1.03–1.17	0.006*
CAD	1.00	0.94–1.05	0.921	0.98	0.92–1.04	0.446
COPD	1.12	1.06–1.18	<0.001*	0.92	0.85–0.99	0.020*
Smoking-related disorder	1.23	1.16–1.31	<0.001*	1.09	1.00–1.19	0.045*
**CT/RT**						
CT+RT	1.10	1.00–1.20	0.052	0.95	0.86–1.04	0.266
CT	0.97	0.88–1.07	0.597	0.85	0.77–0.94	0.002*
RT	1.34	1.18–1.52	<0.001*	1.20	1.06–1.36	0.005*
EGFR-TKI Response						
Responder	0.40	0.38–0.42	<0.001	0.40	0.38–0.42	<0.001*
CT regimens before EGFR-TKI						
≥2	1.08	1.03–1.14	0.002	0.92	0.87–0.97	0.002*

“*”denotes p-value < 0.05. Risks of death are referenced to non-user in statin use, to female insex, to age <65 in age, to low urbanization in urbanization, to 0 in income, to without comorbidities in comorbidities, to without RT or CT in CT/RT, to non-responder in EGFR-TKI response, and to ≤1 in CT regimen before EGFR-TKI.

After adjustment for statin use, age, sex, urbanization, income, DM, hypertension, stroke, CAD, COPD, smoking-related disorders, CT/RT, EGFR-TKI response, and regimens before EGFR-TKI therapy, an increased risk of death was observed in male patients with lung cancer (HR: 1.28, 95% CI: 1.21–1.35, p <0.001), DM (HR: 1.17, 95% CI: 1.10–1.24, p < 0.001), stroke (HR: 1.10, 95% CI: 1.03–1.17, p = 0.006), and RT (HR: 1.20, 95% CI: 1.06–1.36, p = 0.005). A reduced risk of death was still observed in statin users (HR: 0.58, 95% CI: 0.54–0.62, p < 0.001), those with monthly income ≥25000 NT$ (HR: 0.82, 95% CI: 0.76–0.90, p <0.001), CT (HR: 0.85, 95% CI: 0.77–0.94, p = 0.002), EGFR-TKI responder (HR: 0.40, 95% CI: 0.38–0.42, p < 0.001), and ≥2 CT regimens before EGFR-TKI (HR: 0.92, 95% CI: 0.87–0.97, p = 0.002) (Table 2).

The PS-based selection process identified 1918 statin users and 3836 non-users in our study (S1 Table). After PS matching and adjustment for statin use, age, sex, urbanization, income, DM, hypertension, stroke, CAD, COPD, smoking-related disorders, CT/RT, EGFR-TKI response, and regimens before EGFR-TKI therapy (S2 Table), a reduced risk of death was still observed in statin users (HR: 0.61, 95% CI: 0.57–0.65, p < 0.001), those with monthly income ≧25000 NT$ (HR: 0.78, 95% CI: 0.70–0.87, p < 0.001), EGFR-TKI responder (HR: 0.41, 95% CI: 0.38–0.43, p < 0.001), and ≥2 CT regimens before EGFR-TKI (HR: 0.91, 95% CI: 0.85–0.97, p = 0.004) (S2 Table). An increased risk of death was observed in male patients with patients with lung cancer (HR: 1.27, 95% CI: 1.19–1.36, p < 0.001), age ≥65 years (HR: 1.09, 95% CI: 1.01–1.18, p = 0.032), DM (HR: 1.18, 95% CI: 1.11–1.26, p < 0.001), hypertension (HR: 1.10, 95% CI: 1.01–1.20, p = 0.030), stroke (HR: 1.13, 95% CI: 1.05–1.21, p = 0.001), and RT (HR: 1.20, 95% CI: 1.03–1.40, p = 0.019).

### PFS and OS in the statin and non-statin EGFR-TKI lung cancer cohorts

**Fig 2:**
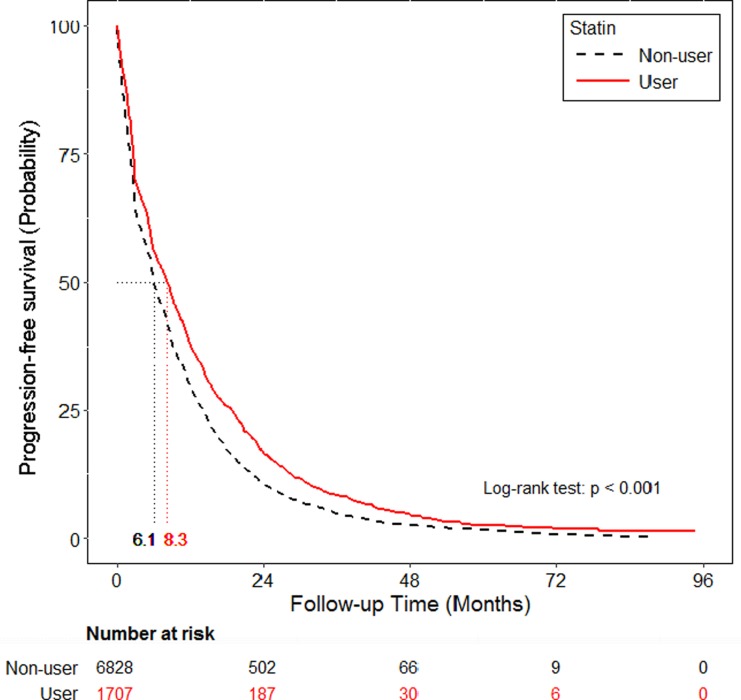
Progression-free survival curve of the statin and non-statin cohorts in patients with EGFR-TKIs lung cancer.

**Fig 3:**
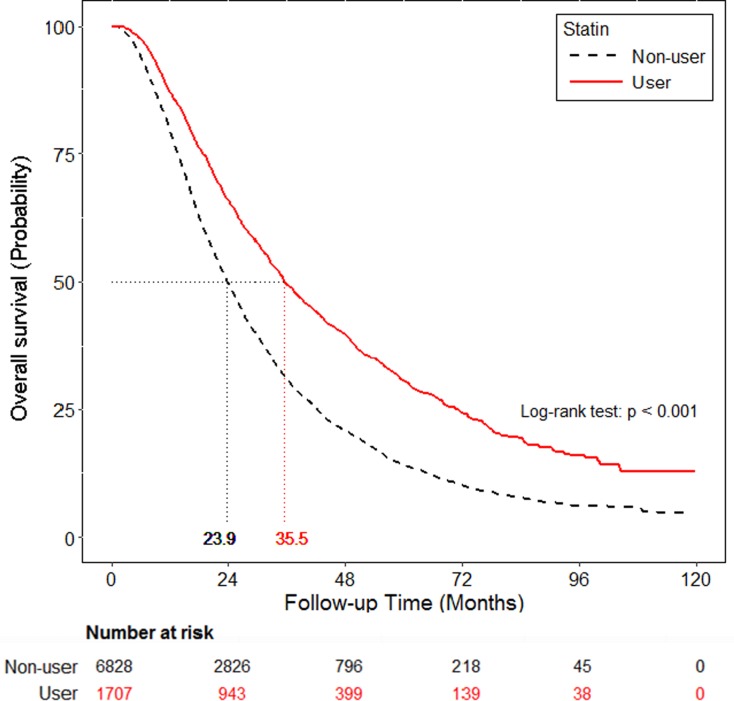
Overall survival curve of the statin and non-statin cohorts in patients with EGFR-TKIs lung cancer.

PFS and OS were also compared among statin users and non-users. Statin use was associated with a significantly longer median PFS with 2^nd^ line EGFR-TKI therapy (8.3 months, 95% CI: 7.6–8.9 vs. 6.1 months, 95% CI: 6.0–6.4, p < 0.001) (Fig 2). Statin use was also associated with a significantly longer median OS (35.5 months, 95% CI: 33.8–38.1 vs. 23.9 months, 95% CI: 23.4–24.7, p < 0.001) (Fig 3).

### HRs of death in subpopulations treated with statin

The effect of statins in reducing the risk of death was then evaluated in subpopulations of lung cancer patients with EGFR-TKI therapy. All subpopulations of statin users stratified by sex, age, DM, hypertension, stroke, CAD, COPD, smoking-related disorder, CT/RT, EGFR-TKIs, EGFR-TKI response, and CT regimens before EGFR-TKI had a significantly reduced risk of death (p < 0.001) (Table 3). Similar results were also observed in the PS-matched groups (S3 Table).

**Table 3:** Adjusted HRs of death in subpopulations treated with statin.

Variable	Statin user					
	Total	Death	(%)	HR*	95% CI	p-value
**Sex**						
Female	4,390	2,895	(65.9%)	0.55	0.50–0.61	<0.001*
Male	4,145	3,160	(76.2%)	0.60	0.55–0.66	<0.001*
**Age (years)**						
<65	3,465	2,437	(70.3%)	0.53	0.48–0.59	<0.001*
≥65	5,070	3,618	(71.4%)	0.60	0.55–0.66	<0.001*
**DM**						
Without	6,036	4,289	(71.1%)	0.51	0.46–0.56	<0.001*
With	2,499	1,766	(70.7%)	0.66	0.59–0.73	<0.001*
**Hypertension**						
Without	3,399	2,498	(73.5%)	0.47	0.40–0.54	<0.001*
With	5,136	3,557	(69.3%)	0.62	0.57–0.67	<0.001*
**Stroke**						
Without	6,758	4,782	(70.8%)	0.56	0.51–0.61	<0.001*
With	1,777	1,273	(71.6%)	0.63	0.55–0.71	<0.001*
**CAD**						
Without	6,014	4,301	(71.5%)	0.53	0.49–0.59	<0.001*
With	2,521	1,754	(69.6%)	0.65	0.58–0.72	<0.001*
**COPD**						
Without	5,833	4,058	(69.6%)	0.55	0.50–0.60	<0.001*
With	2,702	1,997	(73.9%)	0.62	0.55–0.70	<0.001*
**Smoking-related disorder**						
Without	6,812	4,752	(69.8%)	0.56	0.52–0.61	<0.001
With	1,723	1,303	(75.6%)	0.62	0.54–0.72	<0.001
**CT/RT**						
CT+RT	4,270	3,423	(80.2%)	0.59	0.54–0.65	<0.001*
CT	2,495	1,670	(66.9%)	0.59	0.52–0.68	<0.001*
RT	666	434	(65.2%)	0.53	0.39–0.71	<0.001*
Without CT or RT	1,104	528	(47.8%)	0.52	0.42–0.66	<0.001*
**EGFR-TKI**						
Gefitinib	4,200	2,795	(66.5%)	0.53	0.47–0.59	<0.001*
Erlotinib	3,412	2,707	(79.3%)	0.62	0.56–0.69	<0.001*
Both	923	553	(59.9%)	0.60	0.48–0.76	<0.001*
**EGFR-TKI Response**						
Responder	5,069	3,064	(60.4%)	0.58	0.53–0.64	<0.001*
Non-responder	3,466	2,991	(86.3%)	0.59	0.53–0.65	<0.001*
**CT regimens before EGFR-TKI**						
**≤1**	5,528	3,507	(63.4%)	0.60	0.54–0.65	<0.001*
Gefitinib (adenocarcinoma)	3,799	2,229	(58.7%)	0.55	0.49–0.61	<0.001*
Erlotinib (adenocarcinoma)	1,729	1,278	(73.9%)	0.70	0.61–0.81	<0.001*
≥**2**	3,007	2,548	(84.7%)	0.55	0.49–0.61	<0.001*
Gefitinib (adenocarcinoma)	1,197	1,047	(87.5%)	0.51	0.43–0.61	<0.001*
Erlotinib (NSCLC)	1,810	1,501	(82.9%)	0.57	0.50–0.66	<0.001*

“*” denotes p < 0.05. Risks of death are referenced to statin non-users.

## Discussion

In this retrospective, nationwide, longitudinal cohort study, statin use was associated with prolonged PFS and OS in patients with lung cancer receiving EGFR-TKI therapy. Further analysis of subpopulations showed that statin use reduced the risk of death in these patients. For the first time, this nationwide population-based study showed the benefit of statin use in patients with lung cancer receiving EGFR-TKI therapy. A novel method of estimating PFS in NHIRD was also developed in our study.

The underlying mechanism of how statins enhance the effect of EGFR-TKIs in lung cancer cells and patients remains unclear. Statins overcome gefitinib resistance in *in vitro* studies. Both lovastatin [27] and atorvastatin [28] overcame gefitinib resistance in NSCLC cells with *K-Ras* mutations through down-regulation of RAS protein, which leads to inhibition of both the RAF/ERK and AKT pathways. In addition, simvastatin overcame gefitinib resistance in T790M mutant NSCLC cells via an AKT/β-catenin signaling-dependent down-regulation of survivin and apoptosis induction [21]. However, further studies with different EGFR-TKIs (e.g. erlotinib or afatinib) and EGFR-TKI sensitive lung cancer cells are still advocated to elucidate further the underlying interaction mechanism of EGFR-TKIs and statins.

The combination of statin and EGFR-TKI in patients with lung cancer was also beneficial in clinical studies. Simvastatin plus gefitinib improved the response rate and PFS in patients with EGFR wild-type non-adenocarcinoma lung cancer [29]. In another study, the combination of statins (atorvastatin or simvastatin) with EGFR-TKIs (erlotinib or gefitinib) prolonged PFS in NSCLC harboring *KRAS* mutations [22]. Previous studies are limited by small case numbers. To date, our study has the largest case number to show that the combination of statins with EGFR-TKIs prolongs PFS and OS in patients with lung cancer.

In our study, increased risks of death were observed in male patients, old age (> 65 years old), DM, stroke, and radiotherapy. These findings are similar to those of previous studies. Male sex has a negative impact on the outcome of EGFR-TKI therapy compared to female sex [30]. In addition, age might limit chemotherapy use for elderly patients with NSCLC after EGFR-TKIs failure [31]. DM is associated with poor prognosis in patients with lung cancer [32]. An increased risk of stroke was reported in patients with lung cancer [33]. The administration of radiotherapy might imply a more advanced stage of lung cancer, such as brain or bone metastasis. The prognosis of these patients is still poor with a median survival of less than one year [34]. There is a negative association between income and survival of patients with lung cancer [35–37], and our study showed similar results. Factors that might influence the prognosis of cancers, such as advanced tumor stage at diagnosis, behavior in seeking medical care, and smoking status are associated with low socioeconomic status [38].

The status of *EGFR* mutation is not available in the NHIRD. As a result, our studies include patients with lung cancer with wild-type and mutant *EGFR*. We thus alternatively used EGFR-TKI responders as a surrogate for *EGFR* mutations, which are associated with a favorable response to EGFR-TKI therapy. EGFR-TKI responders and non-responders had reduced risks of death, which might imply that statins could have protective effects in lung patients with wild-type and mutant *EGFR*. The PFS in our study is similar to the overall PFS of a previous EGFR-TKI study including patients with both wild-type and mutant *EGFR* [10]. The OS in first-line treatment of patients with EGFR mutation-positive advanced NSCLC ranged from 23.6 to 30.5 months in previous studies [11, 39]. In our study, statin users had a prolonged OS up to 33.8 months. This finding might imply that statins also enhance the therapeutic effects of other treatments such as chemotherapy or radiotherapy or that statins have anti-cancer effects, which was further confirmed in the subpopulation analysis in our study. Recently, a study focusing on lung patients with dyslipidemia showed that statins reduce lung cancer mortality [19], and consistent results were observed in our study. Smoking status is also not available in the NHIRD, and this might also limit the strength of our study.

Statins had a protective effect in patients with lung cancer in both smokers and non-smokers in a large case-control study [40]. In Taiwan, there are fewer female (10%) than male (86%) smokers among patients with lung cancer [41]. Statins showed protective effects in both groups of patients in the stratified analysis of our study. Furthermore, patients with and without smoking-related disorders had reduced risks of death in our study.

Most patients in our study are lung adenocarcinoma patients, and statin use was associated with a reduced the risk of death among these patients. A subgroup of patients with NSCLC, who received ≥2 CT regimens before erlotinib therapy, was also included in our study. Statins also showed protective effects in this subgroup of patients. As a result, statins might have protective effects in lung adenocarcinoma and other patients with NSCLC. However, further prospective studies are warranted.

There are other limitations in this study. Data including lung cancer staging, pathology, cancer related symptoms, physical status, personal characteristics, and environmental or genetic factors were not available in the NHIRD. Patient compliance with anti-cancer therapy was also not available in the database. All of these potential confounders might be associated with the outcome of patients with lung cancer. Using EGFR-TKI responders as a surrogate for activating *EGFR* mutations might have some bias. In addition, even with similar findings in the subgroup analysis, the results of the present study might be confounded by smoking. However, using a nationwide database, a large sample size was derived and adequate statistical power was provided to examine the association between statins and mortality in patients with EGFR-TKI lung cancer. The PS analysis showed a survival benefit of statins when known confounding factors were matched.

In conclusion, our study showed that the use of statins potentially enhances the therapeutic effect and decreases mortality in patients with lung cancer receiving EGFR-TKIs therapy. Prospective randomized controlled trials and further mechanistic studies are needed further to verify our findings.

## Supporting information

S1 Table Characteristics for EGFR-TKIs NSCLC patients after propensity adjustment. (DOCX)

S2 Table Comparison of HRs of death with clinical variables after propensity adjustment. (DOCX)

S3 Table Adjusted HRs of death in subpopulations treated with statin after propensity adjustment. (DOCX)
