# Supplementary material for: Statin improves survival in patients with EGFR-TKI lung cancer: A nationwide population-based study
Source: PLoS One. 2017 Feb 3;12(2):e0171137. doi: 10.1371/journal.pone.0171137 (PMC5291515; doi:10.1371/journal.pone.0171137)
Supplement: S1 Table — (DOCX) [file pone.0171137.s001.docx]

**S1 Table. Characteristics for EGFR-TKIs NSCLC patients after propensity adjustment**

|  | Statin | | | | |
| --- | --- | --- | --- | --- | --- |
| Variables | User | | Non-user | | p-value |
| **Patients** | 1,918 | (100.0%) | 3,836 | (100.0%) |  |
| **Gender** |  |  |  |  | 0.955 |
| Female | 1,023 | (53.3%) | 2,043 | (53.3%) |  |
| Male | 895 | (46.7%) | 1,793 | (46.7%) |  |
| **Age** |  |  |  |  | 0.563 |
| 40-64 | 724 | (37.7%) | 1,418 | (37.0%) |  |
| ≧65 | 1,194 | (62.3%) | 2,418 | (63.0%) |  |
| Mean (SD) | 67.3 | (9.8 ) | 67.7 | (10.8 ) | 0.093 |
| **Urbanization** |  |  |  |  | 0.327 |
| Very high | 641 | (33.4%) | 1,206 | (31.4%) |  |
| High | 795 | (41.4%) | 1,641 | (42.8%) |  |
| Morderate | 313 | (16.3%) | 670 | (17.5%) |  |
| Low | 169 | (8.8%) | 319 | (8.3%) |  |
| **Income (NT$)** |  |  |  |  | 0.002^*^ |
| 0 | 724 | (37.7%) | 1,376 | (35.9%) |  |
| 1-15840 | 336 | (17.5%) | 693 | (18.1%) |  |
| 15841-25000 | 520 | (27.1%) | 1,196 | (31.2%) |  |
| ≧25000 | 338 | (17.6%) | 571 | (14.9%) |  |
| **Comorbidities** |  |  |  |  |  |
| DM | 1,020 | (53.2%) | 2,003 | (52.2%) | 0.490 |
| Hypertension | 1,523 | (79.4%) | 3,142 | (81.9%) | 0.022^*^ |
| Stroke | 576 | (30.0%) | 1,143 | (29.8%) | 0.855 |
| CAD | 890 | (46.4%) | 1,759 | (45.9%) | 0.695 |
| COPD | 662 | (34.5%) | 1,313 | (34.2%) | 0.829 |
| Smoking related disorder | 410 | (21.4%) | 816 | (21.3%) | 0.927 |
| **CT/RT** |  |  |  |  | 0.864 |
| CT+ RT | 882 | (46.0%) | 1,730 | (45.1%) |  |
| CT | 595 | (31.0%) | 1,232 | (32.1%) |  |
| RT | 138 | (7.2%) | 276 | (7.2%) |  |
| Without CT or RT | 303 | (15.8%) | 598 | (15.6%) |  |
| **EGFR-TKI** |  |  |  |  | 0.749 |
| Gefitinib | 945 | (49.3%) | 1,877 | (48.9%) |  |
| Erlotinib | 751 | (39.2%) | 1,535 | (40.0%) |  |
| Both | 222 | (11.6%) | 424 | (11.1%) |  |
| **EGFR-TKI Response** |  |  |  |  | 0.758 |
| Responder | 1,212 | (63.2%) | 2,408 | (62.8%) |  |
| Non-responder | 706 | (36.8%) | 1,428 | (37.2%) |  |
| **CT regimens before EGFR-TKI** |  |  |  |  | 0.844 |
| ≤1 | 1,264 | (65.9%) | 2,518 | (65.6%) |  |
| ≥2 | 654 | (34.1%) | 1,318 | (34.4%) |  |

^“*”^denotes p-value < 0.05.
