# Supplementary material for: Statin improves survival in patients with EGFR-TKI lung cancer: A nationwide population-based study
Source: PLoS One. 2017 Feb 3;12(2):e0171137. doi: 10.1371/journal.pone.0171137 (PMC5291515; doi:10.1371/journal.pone.0171137)
Supplement: S2 Table — (DOCX) [file pone.0171137.s002.docx]

**S2 Table. Comparison of HRs of death with clinical variables after propensity adjustment**

|  | Crude | | | | Adjusted | | | |
| --- | --- | --- | --- | --- | --- | --- | --- | --- |
| Variable | HR | 95% CI | p-value | | HR | 95% CI | p-value | |
| **Statin use** |  |  |  |  |  |  |  |  |
| User | 0.63 | 0.59-0.67 |  | <0.001^*^ | 0.61 | 0.57-0.65 |  | <0.001^*^ |
| **Gender** |  |  |  |  |  |  |  |  |
| Male | 1.36 | 1.28-1.45 |  | <0.001^*^ | 1.27 | 1.19-1.36 |  | <0.001^*^ |
| **Age** |  |  |  |  |  |  |  |  |
| ≧65 | 1.26 | 1.18-1.35 |  | <0.001^*^ | 1.09 | 1.01-1.18 |  | 0.032^*^ |
| **Urbanization** |  |  |  |  |  |  |  |  |
| Very high | 0.93 | 0.82-1.04 |  | 0.208 | 0.93 | 0.81-1.05 |  | 0.237 |
| High | 1.01 | 0.89-1.13 |  | 0.917 | 1.00 | 0.89-1.13 |  | 0.959 |
| Moderate | 1.10 | 0.97-1.25 |  | 0.148 | 1.05 | 0.93-1.20 |  | 0.429 |
| **Income (NT $)** |  |  |  |  |  |  |  |  |
| 1-15840 | 1.05 | 0.96-1.14 |  | 0.321 | 0.91 | 0.84-1.00 |  | 0.054 |
| 15841-25000 | 1.07 | 0.99-1.15 |  | 0.105 | 0.99 | 0.91-1.08 |  | 0.821 |
| ≧25000 | 0.75 | 0.69-0.83 |  | <0.001^*^ | 0.78 | 0.70-0.87 |  | <0.001^*^ |
| **Comorbidities** |  |  |  |  |  |  |  |  |
| DM | 1.30 | 1.22-1.39 |  | <0.001^*^ | 1.18 | 1.11-1.26 |  | <0.001^*^ |
| Hypertension | 1.26 | 1.17-1.37 |  | <0.001^*^ | 1.10 | 1.01-1.20 |  | 0.030^*^ |
| Stroke | 1.22 | 1.14-1.30 |  | <0.001^*^ | 1.13 | 1.05-1.21 |  | 0.001^*^ |
| CAD | 1.15 | 1.09-1.23 |  | <0.001^*^ | 1.05 | 0.98-1.12 |  | 0.144 |
| COPD | 1.15 | 1.08-1.23 |  | <0.001^*^ | 0.97 | 0.89-1.06 |  | 0.463 |
| Smoking related disorder | 1.22 | 1.13-1.31 |  | <0.001^*^ | 1.02 | 0.93-1.13 |  | 0.659 |
| **CT/RT** |  |  |  |  |  |  |  |  |
| CT+RT | 1.05 | 0.95-1.17 |  | 0.363 | 1.03 | 0.92-1.15 |  | 0.633 |
| CT | 0.97 | 0.87-1.09 |  | 0.634 | 0.90 | 0.80-1.01 |  | 0.071 |
| RT | 1.40 | 1.21-1.63 |  | <0.001^*^ | 1.20 | 1.03-1.40 |  | 0.019^*^ |
| **EGFR-TKI Response** |  |  |  |  |  |  |  |  |
| Responder | 0.41 | 0.38-0.43 |  | <0.001^*^ | 0.41 | 0.38-0.43 |  | <0.001^*^ |
| **CT regimens before EGFR-TKI** |  |  |  |  |  |  |  |  |
| ≥2 | 1.02 | 0.96-1.09 |  | 0.456 | 0.91 | 0.85-0.97 |  | 0.004^*^ |

^“*”^denotes p-value < 0.05. Risks of death are referenced to non-user in statin use, to female in gender, to age < 65 in age, to low urbanization in urbanization, to 0 in income, to without comorbidities in comorbidities, to without RT or CT in CT/RT, to non-responder in EGFR-TKI response, and to ≤1 in CT regimen before EGFR-TKI.
