# Supplementary material for: Statin improves survival in patients with EGFR-TKI lung cancer: A nationwide population-based study
Source: PLoS One. 2017 Feb 3;12(2):e0171137. doi: 10.1371/journal.pone.0171137 (PMC5291515; doi:10.1371/journal.pone.0171137)
Supplement: S3 Table — (DOCX) [file pone.0171137.s003.docx]

**S3 Table. Adjusted HRs of death in subpopulations treated with statin after propensity adjustment**

| Variable | Statin User | | | | | | |
| --- | --- | --- | --- | --- | --- | --- | --- |
|  | Total | Death | (%) | HR* | 95% CI | | p-value |
| **Gender** |  |  |  |  |  |  |  |
| Female | 3,066 | 1,960 | (63.9%) | 0.59 | 0.53-0.65 |  | <0.0001^*^ |
| Male | 2,688 | 2,022 | (75.2%) | 0.68 | 0.61-0.74 |  | <0.0001^*^ |
| **Age (years)** |  |  |  |  |  |  |  |
| <65 | 2,142 | 1,437 | (67.1%) | 0.61 | 0.55-0.69 |  | <0.0001^*^ |
| ≧65 | 3,612 | 2,545 | (70.5%) | 0.65 | 0.60-0.71 |  | <0.0001^*^ |
| **DM** |  |  |  |  |  |  |  |
| Without | 2,731 | 1,803 | (66.0%) | 0.57 | 0.52-0.64 |  | <0.0001^*^ |
| With | 3,023 | 2,179 | (72.1%) | 0.68 | 0.62-0.75 |  | <0.0001^*^ |
| **Hypertension** |  |  |  |  |  |  |  |
| Without | 1,089 | 732 | (67.2%) | 0.52 | 0.45-0.61 |  | <0.0001^*^ |
| With | 4,665 | 3,250 | (69.7%) | 0.67 | 0.62-0.72 |  | <0.0001^*^ |
| **Stroke** |  |  |  |  |  |  |  |
| Without | 4,035 | 2,753 | (68.2%) | 0.63 | 0.58-0.68 |  | <0.0001^*^ |
| With | 1,719 | 1,229 | (71.5%) | 0.64 | 0.57-0.73 |  | <0.0001^*^ |
| **CAD** |  |  |  |  |  |  |  |
| Without | 3,105 | 2,121 | (68.3%) | 0.60 | 0.54-0.66 |  | <0.0001^*^ |
| With | 2,649 | 1,861 | (70.3%) | 0.68 | 0.62-0.75 |  | <0.0001^*^ |
| **COPD** |  |  |  |  |  |  |  |
| Without | 3,779 | 2,558 | (67.7%) | 0.61 | 0.56-0.66 |  | <0.0001^*^ |
| With | 1,975 | 1,424 | (72.1%) | 0.68 | 0.61-0.76 |  | <0.0001^*^ |
| **Smoking related disorder** |  |  |  |  |  |  |  |
| Without | 4,528 | 3,080 | (68.0%) | 0.61 | 0.57-0.66 |  | <0.0001^*^ |
| With | 1,226 | 902 | (73.6%) | 0.69 | 0.60-0.80 |  | <0.0001^*^ |
| **CT/RT** |  |  |  |  |  |  |  |
| CT+RT | 2,612 | 2,040 | (78.1%) | 0.66 | 0.60-0.72 |  | <0.0001^*^ |
| CT | 1,827 | 1,242 | (68.0%) | 0.64 | 0.57-0.73 |  | <0.0001^*^ |
| RT | 414 | 276 | (66.7%) | 0.49 | 0.37-0.64 |  | <0.0001^*^ |
| Without CT or RT | 901 | 424 | (47.1%) | 0.59 | 0.48-0.73 |  | <0.0001^*^ |
| **EGFR-TKI** |  |  |  |  |  |  |  |
| Gefitinib | 2,822 | 1,829 | (64.8%) | 0.56 | 0.51-0.62 |  | <0.0001^*^ |
| Erlotinib | 2,286 | 1,769 | (77.4%) | 0.72 | 0.65-0.80 |  | <0.0001^*^ |
| Both | 646 | 384 | (59.4%) | 0.62 | 0.50-0.77 |  | <0.0001^*^ |
| **EGFR-TKI Response** |  |  |  |  |  |  |  |
| Responder | 3,620 | 2,155 | (59.5%) | 0.62 | 0.57-0.68 |  | <0.0001^*^ |
| Non-responder | 2,134 | 1,827 | (85.6%) | 0.61 | 0.55-0.67 |  | <0.0001^*^ |
| **CT regimens before EGFR-TKI** |  |  |  |  |  |  |  |
| **≤1 Total** | 3,782 | 2,360 | (62.4%) | 0.64 | 0.58-0.69 |  | <0.0001^*^ |
| Gefitinib (Adenocarcinoma) | 2,592 | 1,485 | (57.3%) | 0.58 | 0.51-0.64 |  | <0.0001^*^ |
| Erlotinib (Adenocarcinoma) | 1,190 | 875 | (73.5%) | 0.77 | 0.67-0.89 |  | 0.0005^*^ |
| **≥2 Total** | 1,972 | 1,622 | (82.3%) | 0.63 | 0.56-0.70 |  | <0.0001^*^ |
| Gefitinib (Adenocarcinoma) | 787 | 675 | (85.8%) | 0.57 | 0.48-0.68 |  | <0.0001^*^ |
| Erlotinib (NSCLC) | 1,185 | 947 | (79.9%) | 0.66 | 0.57-0.76 |  | <0.0001^*^ |

“*” denotes p<0.05
